# Dietary Inflammatory Potential and Bone Outcomes in Midwestern Post-Menopausal Women

**DOI:** 10.3390/nu15194277

**Published:** 2023-10-07

**Authors:** Mariah Kay Jackson, Laura D. Bilek, Nancy L. Waltman, Jihyun Ma, James R. Hébert, Sherry Price, Laura Graeff-Armas, Jill A. Poole, Lynn R. Mack, Didier Hans, Elizabeth R. Lyden, Corrine Hanson

**Affiliations:** 1 Medical Nutrition, Department of Medical Sciences, College of Allied Health Professions, University of Nebraska Medical Center, Omaha, NE 68198, USA; mariah.jackson@unmc.edu; 2 Physical Therapy, Department of Health and Rehabilitation Sciences, College of Allied Health Professions, University of Nebraska Medical Center, Omaha, NE 68198, USA; 3 College of Nursing, University of Nebraska Medical Center, Lincoln, NE 68508, USA; 4 Department of Biostatistics, College of Public Health, University of Nebraska Medical Center, Omaha, NE 68198, USA; 5 Department of Epidemiology and Biostatistics and Cancer Prevention and Control Program, University of South Carolina, Columbia, SC 29208, USA; 6 Division of Diabetes, Endocrine & Metabolism, Department of Internal Medicine, College of Medicine, University of Nebraska Medical Center, Omaha, NE 68198, USA; 7 Division of Allergy and Immunology, Department of Internal Medicine, College of Medicine, University of Nebraska Medical Center, Omaha, NE 68198, USA; 8 Interdisciplinary Center of Bone Diseases, Bone and Joint Department, Lausanne University Hospital, Lausanne University, 1015 Lausanne, Switzerland

**Keywords:** dietary inflammatory index, post-menopausal women, bone mineral density, trabecular bone scores

## Abstract

Little is known about the inflammatory potential of diet and its relation to bone health. This cross-sectional study examined the association between the inflammatory potential of diet and bone-related outcomes in midwestern, post-menopausal women enrolled in the Heartland Osteoporosis Prevention Study (HOPS) randomized controlled trial. Dietary intake from the HOPS cohort was used to calculate Dietary Inflammatory Index (DII^®^) scores, which were energy-adjusted (E-DII^TM^) and analyzed by quartile. The association between E-DII and lumbar and hip bone mineral density (BMD) and lumbar trabecular bone scores (TBS; bone structure) was assessed using ANCOVA, with pairwise comparison to adjust for relevant confounders (age, education, race/ethnicity, smoking history, family history of osteoporosis/osteopenia, BMI, physical activity, and calcium intake). The cohort included 272 women, who were predominately white (89%), educated (78% with college degree or higher), with a mean BMI of 27 kg/m^2^, age of 55 years, and E-DII score of −2.0 ± 1.9 (more anti-inflammatory). After adjustment, E-DII score was not significantly associated with lumbar spine BMD (*p* = 0.53), hip BMD (*p* = 0.29), or TBS at any lumbar location (*p* > 0.05). Future studies should examine the longitudinal impact of E-DII scores and bone health in larger, more diverse cohorts.

## 1. Introduction

As the most common bone disease among adults, osteoporosis is a major public health concern, affecting approximately 54 million people in the United States and 200 million worldwide [1,2]. Osteoporosis increases risk of fractures, leading to increased disability, decreased mobility, and decreased quality of life [2,3]. Post-menopausal women are a high-risk group for developing osteoporosis as the hormonal changes surrounding menopause contribute to rapid bone loss, creating porous and thin bones [3]. This leads to one in two post-menopausal women developing a fracture [1,2]. Testing bone mineral density (BMD) is a widely accepted marker for the diagnosis of osteoporosis and can aid in the prediction of fracture risks. Dual-energy X-ray absorptiometry (DXA) is a validated method of assessing bone health outcomes [1,2]. Trabecular bone scores (TBS), used in DXA measurements to assess bone microarchitecture for variation in bone texture, are an emerging adjunct fracture risk identifier [4,5]. As such, TBS provide additional bone health information on bone structure, complementing the standard BMD assessment and enhancing fracture risk stratification [5]. Therefore, it is pertinent to include measures of TBS in the evaluation of overall bone health, something not currently seen in the majority of bone health literature.

There are many lifestyle modifications, including diet, known to aid in the prevention and management of osteoporosis. Traditional bone-related nutrients including vitamins D and K, calcium, and magnesium, are known to play significant roles in the protection and promotion of bone health [6]. Most commonly, these nutrients are found in dairy products, which has received thorough investigation in terms of its role in increasing BMD in post-menopausal women [7]. Furthermore, many studies have examined the effects of specific dietary patterns including the Mediterranean diet, Healthy Eating Index, and Alternate Healthy Eating Index, among others, on bone health and osteoporosis [8,9]. Dietary patterns that are high in vegetables, fruits, fish, whole grains, legumes, and dairy products are associated with higher BMD, while dietary patterns with the excessive consumption of sweets, caffeinated beverages, and meats are associated with lower BMD [10,11]. However, some research has yielded inconsistent results, with other studies reporting no association between dietary pattern scores and bone density [9,12].

The association between dietary patterns and bone health may also be related to the inflammatory effect of diet on bone health. Chronic inflammation stems from a variety of sources, including diet, and is associated with a deleterious effect on bone health [13,14]. The pro-inflammatory potential of the diet is associated with biomarkers of inflammation including C-reactive protein (CRP), interleukin-1β (IL-1β), IL-6, and tumor necrosis factor-alpha (TNF-α) and anti-inflammatory diet potential is associated with the biomarkers IL-4 and IL-10 [15]. Higher concentrations of these circulating inflammatory biomarkers, including CRP, IL-6, and TNF-α, have been associated with lower BMD [13,16]. A novel approach to studying the effect of inflammatory diets on bone outcomes is the Dietary Inflammatory Index (DII^®^) [15]. As opposed to previous studies that have evaluated certain dietary patterns, the DII itself is not a dietary pattern. Rather, the DII is a scoring algorithm that takes into consideration the synergistic effect of multiple individual nutrients and certain foods (e.g., garlic and onion) on the inflammatory potential of the diet.

While diet plays a key role in modulating inflammation, little research has been performed utilizing a quantitative measure of the overall effect of diet on inflammatory potential and its relation to bone health in the prevention and management of osteoporosis. Thus, the objective of this study was to determine the relationship between the inflammatory potential of diet, as measured by the DII, and bone-related outcomes of midwestern post-menopausal women screened for enrollment in the Heartland Osteoporosis Prevention Study (HOPS) [17]. It was hypothesized that a diet with higher inflammatory potential would be associated with poorer measures of bone health as measured using DXA.

## 2. Materials and Methods

**Figure 1:**
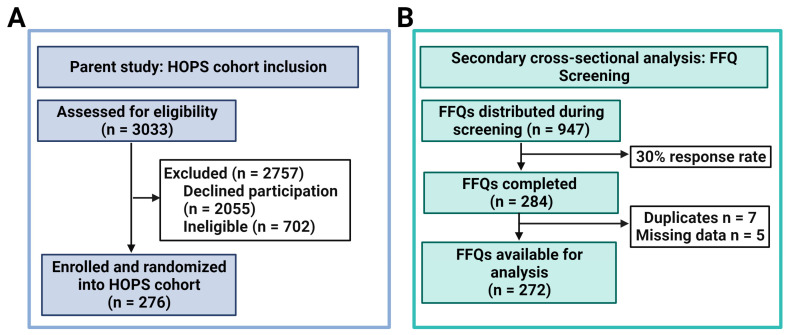
(**A**,**B**). Participant inclusion flowchart for parent study (**A**); Food frequency questionnaire (FFQ) inclusion flowchart (**B**).

Study population: This study was a secondary cross-sectional analysis of data collected from the Heartland Osteoporosis Prevention Study (HOPS). HOPS was a randomized controlled trial (RCT) that evaluated the impact of risedronate versus exercise on bone outcomes in women within six years of menopause with a diagnosis of low bone mass (T-score of −1.0 to −2.49) [17]. Potential participants were recruited from eastern Nebraska and western Iowa and underwent a multi-step screening process, starting with a pre-screen questionnaire. In the parent study, 3033 persons were assessed for eligibility, with a total of 276 out of the optimal enrollment goal of 302 enrolled and randomized (Figure 1A) [18]. For inclusion into the present study, all women completing the in-person screening visit, which included a DXA scan, physical activity assessment, and a demographic questionnaire, were eligible, regardless of the parent study eligibility criteria. The HOPS RCT was approved by the Institutional Review Board (IRB) of the University of Nebraska Medical Center and written consent was obtained at the time of screening. Additional information regarding HOPS recruitment, enrollment, and HOPS study design are detailed elsewhere [17].

Diet Intake Assessment: In addition to the original study protocol, the need for complete dietary assessment was acknowledged. Therefore, a separate protocol was developed to capture these data after the initiation of the parent study. Screening for this ancillary study was performed at the time of eligibility screening after original enrollment began. The present study evaluated dietary intakes using the Harvard semi-quantitative food frequency questionnaire (FFQ), which has been previously validated and used in a variety of cohorts, including female populations [19,20]. Dietary intake was assessed during the screening process, where FFQs were either provided to women at the in-person screening assessment or mailed to their home address. A total of 947 FFQ surveys were distributed. Of those 947 surveys, 284 were completed—a 30% response rate. Of the 284 completed surveys, seven duplicate records were removed (retaining the first entry), and five records were removed for missing data, for a total of 272 eligible diet records (Figure 1B). The completed FFQs were analyzed by the Nutrition Department, Harvard School of Public Health, providing absolute values for intakes of nutrients, including supplements.

Calculation of the Dietary Inflammatory Index (DII^®^): The DII has been previously validated with various inflammatory markers, including in post-menopausal women, and a complete description of the DII scoring process has been detailed elsewhere [15,21]. To summarize, the DII includes up to 45 components with individual inflammatory effect scores. In the present study, a total of 34 DII components were available from the FFQs for score calculation (Table 1). The calculation for the DII is based on dietary data from a world database, providing an estimate of a mean and standard deviation (SD) for each food-derived parameter. Dietary data collected from study participants are used to calculate z-scores and centered percentiles based on the world average intake in order to minimize “right skewing” [15]. The centered proportion scores are multiplied by the corresponding food parameter effect scores, creating food parameter-specific DII scores that are summed to produce the overall DII score for each participant. In this study, energy-adjusted DII (E-DII™) scores were calculated for each participant, using the available assessed nutrients from the FFQs to account for caloric intake in relation to the amount of nutrients consumed. E-DII scores are calculated per 1000 calories of food consumed, utilizing the energy-standardized version of the world database [22]. A higher, more positive E-DII score designates a more pro-inflammatory diet, whereas smaller, more negative E-DII values indicate more anti-inflammatory diets. In this analysis, E-DII scores were the primary exposures. E-DII scores were analyzed by quartiles, wherein the highest E-DII quartile (quartile 4) represents the most pro-inflammatory diet, while the lower quartiles indicate more anti-inflammatory diets.

Bone outcome measures: The outcome measures for this study included bone outcomes from DXA scans, which provide BMD and TBS, the latter being a measure of bone structure. BMD and TBS measurements were taken using a Hologic DXA instrument (Hologic QDR2000™ model, Hologic Inc., Waltham, MA, USA) [17]. The densitometer was operated by a certified radiological technician at Creighton University Osteoporosis Research Center. BMD outcome locations included lumbar spine BMD (L1-L4) and total hip BMD. TBS was obtained via the re-analysis of lumbar spine DXA images, using the beta version 4.0 of TBS software (TBS Osteo 4.0-beta) to account for abdominal soft tissue thickness (TBS iNsight^®^; Medimaps Group USA LLC, Wilmington, DE, USA) [17]. TBS outcome locations included TBS of individual lumbar vertebra (L1, L2, L3, and L4) and total TBS L1–L4.

Other measures: Baseline demographic information and clinical data were obtained from the HOPS database and included age (years), education (some college or less, which includes less than a high school diploma, high school diploma or some college but no degree; college degree; master’s or doctorate degree), race/ethnicity (white/non-Hispanic; non-white/other), smoking history (ever/never), BMI (≤24.9 kg/m^2^, 25–29.9 kg/m^2^, and ≥30 kg/m^2^), calcium intake (including supplemental calcium; mg), and physical activity category. Physical activity was evaluated via the International Physical Activity Questionnaire (IPAQ) and activity levels were categorized into inactive, minimally active, and health-enhancing physical activity (HEPA) [23]. HEPA denotes physical activity levels that exceed the minimum public health physical activity recommendations.

Statistical analysis: Baseline descriptive statistics were reported for all continuous and categorical variables (mean, standard deviation, counts and percentages). The baseline statistics were presented for the total population and by E-DII quartile, with quartile 1 representing the greatest anti-inflammatory potential and quartile 4 representing the least substantial pro-inflammatory potential. Differences in baseline characteristics were assessed by E-DII quartile, with analysis of variance (ANOVA) used for continuous data and chi-square tests applied to categorical data with pairwise adjustments for multiple comparisons. The differences in bone outcomes were assessed by E-DII quartile via ANCOVA, with pairwise adjustments used for multiple comparisons to explore the adjusted relationship addressing covariances of age, education, race/ethnicity, smoking history, family history of osteoporosis/osteopenia, BMI, physical activity, and calcium intake. Analysis was conducted using SAS, Version 9.4. For all analyses, a *p* value < 0.05 was considered statistically significant.

## 3. Results

### 3.1. Characteristics of the Study Population

The final analysis included 272 participants (Figure 1B). Participant characteristics, categorized by quartiles, are displayed in Table 2 according to total population and E-DII scores. The majority of participants were white (89%), never-smokers (76%), and overweight (mean BMI of 27 kg/m^2^), with a mean age of about 55 years old. The mean (SD) E-DII score was −2.0 ± 1.9, indicating more anti-inflammatory diet potential. Baseline mean calcium intake was 1396.4 ± 604.2. There were no significant differences between E-DII quartiles in relation to education level, BMI categories, smoking status, race, and family history of osteoporosis. Calcium intake was significantly different across E-DII quartiles (*p* = 0.01). Physical activity was significantly different across E-DII quartiles (*p* = 0.02), where those in quartile 1 (most anti-inflammatory) had a higher percentage of participants with HEPA (E-DII Q1: 41% HEPA) compared to those in quartile 4 (most pro-inflammatory), who had the highest proportion of inactive participants (E-DII Q4: 44% Inactive).

### 3.2. E-DII Quartiles and Bone Outcomes

Results for the ANCOVA analysis between E-DII quartiles and bone outcomes are displayed in Table 3. No significant associations were found between DXA outcomes and E-DII quartiles after adjustment.

## 4. Discussion

In this study, there was no association between bone outcomes, including BMD, TBS, and dietary inflammatory potential. To our knowledge, we are among the first to include TBS among bone outcomes in the assessment of their relationship with E-DII in post-menopausal women. While chronic inflammation has been associated with an increased risk in osteoporosis and dietary patterns are known to impact inflammatory markers, little investigation has been carried out using E-DII to evaluate the impact on bone health.

Our cohort was predominately white, with most being overweight or obese, and the mean E-DII scores suggested an anti-inflammatory diet. While not all previous studies reported E-DII scores, our population’s average E-DII score was more anti-inflammatory compared to previous DII studies involving post-menopausal cohorts, which had reported higher DII scores ranging from −1.18 to 0.85 [24,25,26,27,28]. This suggests that the diet of our sample was more anti-inflammatory than that of the larger population of post-menopausal women, which could limit our ability to see an association between E-DII and bone outcomes. Additionally, 78% of the sample had completed college or obtained a post-graduate degree. This high level of education likely contributed to diet habits being healthier than those of the broad population, with there being consistent evidence that more highly educated women bias self-reports of their intake towards less calorie-dense foods on the basis of social desirability [29,30,31]. Therefore, it is conceivable that self-reports in this cohort reflect such bias. Unfortunately, as with most studies, no information on social desirability was collected within the HOPS cohort to account for this factor.

TBS has been associated with fracture history and improving prediction of fracture risk, with increasing attention in its role in risk of osteoporosis, but its relationship with dietary factors is yet to be fully understood [4,5]. Of the few studies that have examined the impact of nutrition or dietary patterns on TBS, most report no association with dietary factors such as dairy or calcium intake [32,33,34]. A three-year retrospective medical record review of middle-aged to older women examined the impact of vegetarian dietary patterns on bone outcomes, including BMD and TBS. This study found that, while perimenopausal-aged women following a vegetarian diet had lower BMD, TBS did not differ between dietary patterns for any ages [35]. Other studies provide inconsistent evidence on the impact of alcohol use and protein sources on TBS [36,37,38]. For example, in the VITAL RCT, which included women aged ≥ 55 years, univariate analysis demonstrated that high alcohol intake was associated with lower TBS (*p* = 0.009). However, when adjusted for age, sex and race, alcohol intake was positively associated with TBS (β = 0.019, *p* = 0.041) [37]. This contradicts an earlier study examining Canadian post-menopausal women, which found, after adjustment, alcohol abuse to be associated with lower TBS [38]. There is even further limited evidence on the use of the E-DII/DII and trabecular measurements, with one study of healthy adolescents find no relationship between the DII and tibia trabecular area after adjustment [39]. While our findings are consistent with most of the existing diet-related literature, further investigation is warranted among larger, more diverse cohorts.

Unlike previous studies that have reviewed the DII and bone outcomes, our study did not find any significant association between BMD and E-DII scores after adjustment for important potential confounders. One of the first studies to evaluate the inflammatory potential of diet using the DII and BMD was an observational study of post-menopausal Iranian women aged 50–85 years [40]. After adjustment, an increase in DII was found to be significantly associated with a decrease in the BMD of the lumbar spine (β = −0.03, *p* = 0.001). When the DII was analyzed as a categorical variable, the odds of having a DII score greater than the DII median was 2.3 times higher in those with lower lumbar spine BMD, suggesting that poorer lumbar spine BMD was associated with diets that have greater pro-inflammatory potential (OR = 2.3, *p* = 0.04) [40]. Adding to the literature, a study by Mazidi et al. aimed to evaluate the relationship between the DII, bone fractures, and DXA outcomes [41]. This study was conducted among American men and women using the 2005 and 2010 National Health and Nutrition Examination Surveys (NHANES). In the adjusted, stratified analysis of women, moving from quartile 1 (least inflammatory) to quartile 4 (most inflammatory), we found that those with more anti-inflammatory diet potential had significantly higher mean lumbar vertebrae L3 BMD, lumber vertebrae L4 BMD, total femur BMD, femoral neck BMD, trochanter BMD, and intertrochanter BMD [41]. While our study adjusted for many of the same covariates, the study of Mazidi et al. additionally controlled for C-reactive protein (CRP). This may have affected their outcomes as CRP levels take into account systemic inflammation status. The inflammatory potential of diets has also been shown to impact BMD over time, as seen in a study utilizing the Women’s Health Initiative, one of the largest post-menopausal women cohorts in the United States [25]. Here, they demonstrated how, after adjustment, women with the least substantial inflammatory diet potential (quartile 1) had a less significant loss of hip BMD over a period of six years than those in quartile four with the highest inflammatory potential (*p* < 0.0001). Interestingly, a recent meta-analysis of bone heath and DII found BMD and fracture risk to only be significantly associated with DII scores in non-cohort studies (i.e., case control or cross-sectional) [42]. While our results represent a cross-sectional analysis of an RCT and differ from these recent findings, it is possible that differences in sample size or populations studied, including potential for reporting bias, led to these contrary findings.

There are several potential mechanisms by which diet impacts bone outcomes, including the role of chronic inflammation and oxidative stress [43,44]. In fact, a recent systematic review and meta-analysis of post-menopausal osteoporosis demonstrated that specific oxidative stress biomarkers, accompanied by decreased total antioxidant status and total antioxidant power, were significant among those with osteoporosis. Additionally, it was found that these markers may be beneficial to follow in clinical care [44]. As diet serves as an ample source of antioxidants and has previously been associated with decreasing inflammation [45], it warrants further investigation for its potential to improve bone health. Preliminary research has been undertaken with antioxidant dietary components used to improve bone health in post-menopausal women. Examples include resveratrol, which can be found in grapes and berries. The Resveratrol for Healthy Aging in Women (RESHAW) trial examined the impact of the twice-daily supplementation of resveratrol on bone health in a 24-month randomized crossover controlled intervention [46]. After 12 months, bone density was improved in the intervention group, along with a reduction in the probability of fracture risk. This study highlights the utility of emphasizing the dietary intake of antioxidant-rich foods, such as resveratrol. While individual dietary components may contribute to bone health outcomes, the DII integrates the synergistic effects of multiple antioxidants, nutrients, and foods together, warranting further investigation.

It is also important to note the interplay between diet, obesity, and inflammation as it pertains to bone health. As previously stated, the majority of our population were overweight or obese. Obesity is associated with increased systemic inflammation and oxidative stress, leading to higher risk of fracture and osteoporosis, as well as the dysregulation of calcium homeostasis and protein synthesis [47,48,49,50,51]. Therefore, it is reasonable to speculate that the effect of pro-inflammatory diets on bones could be more pronounced in women who already carry an increased burden of inflammation due to obesity. Few studies have been conducted into defining the connection between higher BMI and pro-inflammatory DII scores with poorer bone health, including the increased risk of fractures and osteoporosis. A study in post-menopausal women based on data from 2009 to 2011 Korea National Health and Nutrition Examination Surveys found that those with DII scores of >−0.07 (more pro-inflammatory) were 2.757 (95% CI: 1.398, 5.438, *p* = 0.004) times more likely to have osteopenic obesity and 2.186 (95% CI: 1.182, 4.044; *p* = 0.013) times more likely to have osteosarcopenic obesity than those with more anti-inflammatory DII scores (≤−0.07) [52]. It is important to note that in this population of Korean women, a BMI of >25kg/m^2^ was used to define obesity, whereas this would encompass overweight and obesity within United States National Institutes of Health (NIH) guideline classifications [53]. This study reinforces the idea that relative components of lean and fat mass are important factors to consider in the relationship of DII and bone outcomes, not just weight status or BMI that are important, as current literature supports lean mass over fat mass use as a stronger predictor of BMD [54]. While our study controlled for BMI status, future studies should include additional measures of body composition.

### Study Limitations

This is a cross-sectional study with DII and bone outcomes only analyzed at one time point, the screening visit of the parent RCT. Therefore, we could not conduct a prospective examination of the effects of the inflammatory potential of diet on bone outcomes. However, this is the first study to our knowledge to examine TBS scores in relation to E-DII. Little research has been undertaken to evaluate the relationship between nutrition and TBS; therefore, this study is important to moving the literature forward. While this study may be generalizable to white, midwestern post-menopausal women, future studies should aim to include more diverse populations. This study used an FFQ to assess dietary intake which can lead to under- or over-estimation of the actual dietary intake of the variables due to participant recall bias. An FFQ response rate of 30% may also carry additional bias in responders compared to non-responders, which cannot unable to be assessed. Additionally, social desirability bias in self-report data may be more strongly expressed in women, especially highly educated women, as seen in our population. This can potentially lead to skewed nutrient intakes from which E-DII scores were calculated [29,30,31]. However, the DII is a robust and validated scoring system used in numerous populations in more than 60 countries around the world [15]. To combat self-report bias and further quantify exposures, future studies should include the concurrent evaluation of biomarkers of nutrient intake and inflammation. Not accounting for vitamin D status at baseline may interfere with the analysis as low vitamin D status may contribute to poor bone outcomes independently. This would be an important covariate to include in future studies due to its role in bone health.

## 5. Conclusions

Osteoporosis is an important health concern, impact the lives of post-menopausal women and healthcare. Diet has long been studied for its potential as a minimally invasive preventative measure and treatment for osteoporosis. Our study advances the current literature as the first to assess the relationship of TBS and E-DII scores within post-menopausal women. However, we did not observe any significant associations between diet-derived inflammation and bone health after adjustment. It will be important for future studies to determine the impact of E-DII scores over time and whether changing to a more anti-inflammatory dietary pattern is associated with better bone outcomes long-term.

## Figures and Tables

**Table 1:** DII components available for calculation of the DII score *.

Alcohol	Energy (kcal)	Niacin	Selenium	Flavan-3-ol
Vitamin B12	Total Fat	*n*-3 fatty acids	Thiamin	Flavones
Vitamin B6	Fiber	*n*-6 fatty acids	Vitamin A	Flavonols
β-Carotene	Folic Acid	Protein	Vitamin C	Flavonones
Caffeine	Iron	PUFA	Vitamin D	Anthocyanidins
Carbohydrate	Magnesium	Riboflavin	Vitamin E	Isoflavones
Cholesterol	MUFA	Saturated Fat	Zinc	

* 34 of 45 components available for calculation. Components not available for calculation: eugenol, garlic, ginger, onion, saffron, turmeric, green/black tea, pepper, thyme/oregano, trans fat, rosemary. Abbreviations: DII: Dietary Inflammatory Index; MUFA: monounsaturated fatty acid; *n*-3 fatty acids: omega-3 fatty acids; *n*-6 fatty acids: omega-6 fatty acids; PUFA: polyunsaturated fatty acids.

**Table 2:** Participant characteristics of post-menopausal women overall and by quartiles of E-DII scores.

Characteristics	Overall (*n* = 272)	Q1 (*n* = 68)	Q2 (*n* = 68)	Q3 (*n* = 68)	Q4 (*n* = 68)	*p* Value *
**Continuous Variables Mean (SD)**						
**E-DII Mean (SD)**	−2.0 ± (1.9)	−4.0 ± (0.5)	−2.8 ± (0.2)	−1.7 ± (0.4)	0.6 ± (1.2)	<0.0001
**Age**, years	54.7 ± 3.3	54.7 ± 3.7	54.8 ± 3.1	54.7 ± 3.4	54.5 ± 3.0	0.74
**Calcium intake**, mg	1396.4 ± 604.2	1584.4 ± 620.2	1384.1 ± 572.7	1219.7 ± 533.0	1378.4 ± 640.7	0.01 ^†^
**Categorical Variables *n* (%)**						
**BMI category** ≤24.9 kg/m^2^ 25–29.9 kg/m^2^ ≥30 kg/m^2^	124 (46) 71 (26) 77 (28)	39 (57) 13 (19) 16 (24)	34 (50) 16 (24) 18 (26)	26 (38) 18 (26) 24 (35)	25 (37) 24 (35) 19 (28)	0.13
**Education** Some college or less College degree Master’s or doctorate	66 (22) 145 (54) 66 (24)	14 (21) 34 (50) 20 (29)	16 (24) 40 (60) 11 (16)	16 (24) 37 (54) 15 (22)	14 (21) 34 (50) 20 (29)	0.62
**Race** White/non-Hispanic Non-white/other	241 (89) 31 (11)	56 (82) 12 (18)	63 (93) 5 (7)	61 (90) 7 (10)	61 (90) 7 (10)	0.27
**Smoking status** Ever smoker Never smoker	56 (24) 176 (76)	18 (30) 43 (70)	12 (20) 49 (80)	11 (21) 41 (79)	15 (26) 43 (74)	0.58
**Family history of osteoporosis**						
Yes No	118 (44) 149 (56)	36 (54) 31 (46)	25 (37) 41 (63)	31 (46) 36 (54)	26 (39) 40 (61)	0.21
**Physical activity** Inactive Minimal HEPA	89 (33) 92 (34) 90 (33)	18 (27) 21 (31) 28 (42)	14 (21) 26 (38) 28 (41)	27 (40) 21 (31) 20 (29)	30 (44) 24 (35) 14 (21)	0.02

* ANOVA with pairwise comparison for continuous variables; chi-square for categorical variables. ^†^ Pairwise comparison shows significant difference between Q1 and Q3, adjusted *p* = 0.007. Abbreviations: E-DII: Energy-adjusted Dietary Inflammatory Index; BMI: body mass index; HEPA: health-enhancing physical activity.

**Table 3:** The relationship between E-DII quartiles and mean bone outcomes of post-menopausal women after adjustment for relevant confounders.

	Q1	Q2	Q3	Q4	*p* Value
Adjusted Mean * ± SE					
Lumbar spine BMD (g/cm^2^)	0.998 ± 0.022	1.025 ± 0.023	0.991 ± 0.023	0.995 ± 0.022	0.53
Total hip BMD (g/cm^2^)	0.913 ± 0.016	0.949 ± 0.017	0.936 ± 0.018	0.930 ± 0.017	0.29
TBS-L1	1.338 ± 0.016	1.360 ± 0.017	1.345 ± 0.017	1.339 ± 0.017	0.65
TBS-L2	1.374 ± 0.015	1.399 ± 0.016	1.370 ± 0.016	1.361 ± 0.016	0.19
TBS-L3	1.374 ± 0.015	1.392 ± 0.016	1.358 ± 0.017	1.353 ± 0.016	0.13
TBS-L4	1.336 ± 0.016	1.330 ± 0.017	1.314 ± 0.017	1.329 ± 0.017	0.69
Total TBS L1-L4	1.000 ± 0.021	1.021 ± 0.022	0.994 ± 0.023	0.997 ± 0.022	0.69

* Least squared (LS) means adjusted for BMI group, age, education, race/ethnicity, smoking status, calcium intake, family history, and physical activity. Abbreviations: E-DII: Energy-adjusted Dietary Inflammatory Index; SE: standard error; Q1–Q4: Quartiles 1–4; BMD: bone mineral density; TBS: trabecular bone score.

## Data Availability

Not applicable.
